# Bacterial domain fusion drives biomineralization innovation in *Colepidae* ciliates

**DOI:** 10.1128/mbio.03654-25

**Published:** 2026-06-03

**Authors:** Keke Wu, Wenyu Chen, Chenghu Fan, Xuqi Lu, Bing Zhang, Miao Miao

**Affiliations:** 1 Medical School, University of Chinese Academy of Sciences 74519, Beijing, China; 2 Department of Rheumatology and Immunology, National Key Laboratory for Immunity and Inflammation, Changzheng Hospital, Naval Medical University 12521, Shanghai, China; 3 Key Laboratory of Systems Health Science of Zhejiang Province, School of Life Science, Hangzhou Institute for Advanced Study 638898, Hangzhou, China; 4 University of Chinese Academy of Sciences, Beijing, Beijing, China; Georgia Institute of Technology, Atlanta, Georgia, USA

**Keywords:** protists, biomineralization, horizontal gene transfer, domain fusion, *Ciliophora*

## Abstract

Mineralized external structures have evolved independently across unicellular eukaryotes. Within the phylum *Ciliophora*, this trait’s restriction to the family *Colepidae* makes it an ideal model for dissecting the genomic basis of this innovation. Here, we assembled high-quality macronuclear genomes for three *Colepidae* species (*Coleps hirtus*, *Levicoleps biwae*, and *Coleps viridis*), and uncovered a marked expansion of gene families implicated in calcium carbonate biomineralization. Phylogenetic analysis reveals that a novel aldo-keto reductase (*Aldo*) domain was horizontally transferred from bacteria to the *Colepidae* lineage. This domain was incorporated into a novel fusion protein exclusive to *Colepidae*, where the N-terminal *Aldo* domain is fused to a canonical carbonic anhydrase (*Carb*) catalytic domain. RNA interference shows that *Carb::Aldo* is required for calcified armor and normal physiology. Together, these findings reveal a previously underappreciated evolutionary route to complex phenotypes in eukaryotes, mediated by bacterial domain fusion and gene-family expansion. This work highlights that subgene-scale horizontal gene transfer (HGT) from bacteria may be an overlooked mechanism driving the evolution of eukaryotic complexity.

**IMPORTANCE:**

Biomineralization is a key ecological trait, yet its genomic basis in early-branching eukaryotes remains largely elusive. Here, we establish the ciliate family *Colepidae* as a tractable genomic model for studying calcium carbonate biomineralization. We reveal that the emergence of their calcified armor coincides with a massive expansion of biomineralization-related gene families and a highly unusual subgene-scale horizontal gene transfer from bacteria. We functionally validated that a novel fusion protein, which combines a co-opted bacterial domain with a eukaryotic catalytic domain, is strictly required for armor synthesis. This study not only illuminates the molecular machinery of ciliate biomineralization but also profoundly reshapes our understanding of evolutionary innovation, demonstrating how the hijacking and repurposing of bacterial genetic fragments can orchestrate complex structural adaptations in eukaryotes.

## INTRODUCTION

Biomineralization is a fundamental biological process that enables organisms to construct protective and supportive structures through the deposition of calcium-based minerals such as calcium carbonate (1, 2). It is widespread across the tree of life: mollusks form shells composed of ~95% calcium carbonate (3), while vertebrates construct mineralized tissues, including bones and teeth in a similar way (4). This process relies on coordinated molecular mechanisms, including calcium ion transport (5), enzymatic production of bicarbonate by carbonic anhydrases (6), and the regulation of mineral crystallization by extracellular matrix proteins (7). Despite its ubiquity in multicellular organisms, biomineralization remains relatively uncommon among unicellular eukaryotes. Only a few lineages, such as planktonic calcifiers like *Radiolaria* (8), are known to possess this capability, and the genomic and molecular mechanisms underlying biomineralization in unicellular eukaryotes remain largely unexplored.

Ciliates (phylum *Ciliophora*) represent one of the most morphologically and genetically diverse groups of unicellular eukaryotes (9–12) and are distinguished by their nuclear dualism: a silent diploid germline micronucleus and a transcriptionally active polyploid macronucleus that functions as the soma. As key model organisms, ciliates offer profound insights into cell biology, genetics, genomics, and ecology (13). This diversity extends to their extracellular coverings, ranging from the proteinaceous cortical membrane in *Paramecium* and *Tetrahymena* to chitin-based armor in *Euplotes*, and to the agglutinated lorica that can be shed in tintinnids (14). In contrast, members of the *Colepidae* (belonging to class *Prostomatea*) develop the most elaborate coverings in the form of permanent, biomineralized calcified shells that are structurally integrated with the somatic cortex.

In *Colepidae,* calcified coverings are composed of distinctive alveolar plates that consist primarily of amorphous calcium carbonate and dicalcium phosphate embedded in a polysaccharide matrix. These structures form a protective armor that shields the cell from environmental stresses, predators, or mechanical damages (15–17). Although morphologically striking and unusually distinct from other ciliate lineages, they resemble the tests of certain foraminifera in mineral composition (18). This convergence points to functional adaptation arising through independent evolutionary routes, yet the genomic basis of this trait remains poorly understood. Addressing these questions is essential for advancing our understanding of how complex structures evolve in unicellular eukaryotes.

Here, we integrate high-quality genomes, comparative and phylogenetic analyses, transcriptomic profiling of shell regeneration, and functional validation via RNA interference to dissect the genomic basis of biomineralization in this lineage. Furthermore, to provide biochemical evidence for the functional coupling between the *Carb* and *Aldo* domains within the identified fusion protein, we perform heterologous expression, purification, and *in vitro* enzyme activity assays. By linking genome evolution to phenotypic adaptation, this study not only establishes *Colepidae* as a model for investigating biomineralization in unicellular eukaryotes but also highlights domain-scale horizontal gene transfer as an overlooked mechanism driving evolutionary innovation.

## RESULTS

### Genomic architecture of *Colepidae*

**Fig 1:**
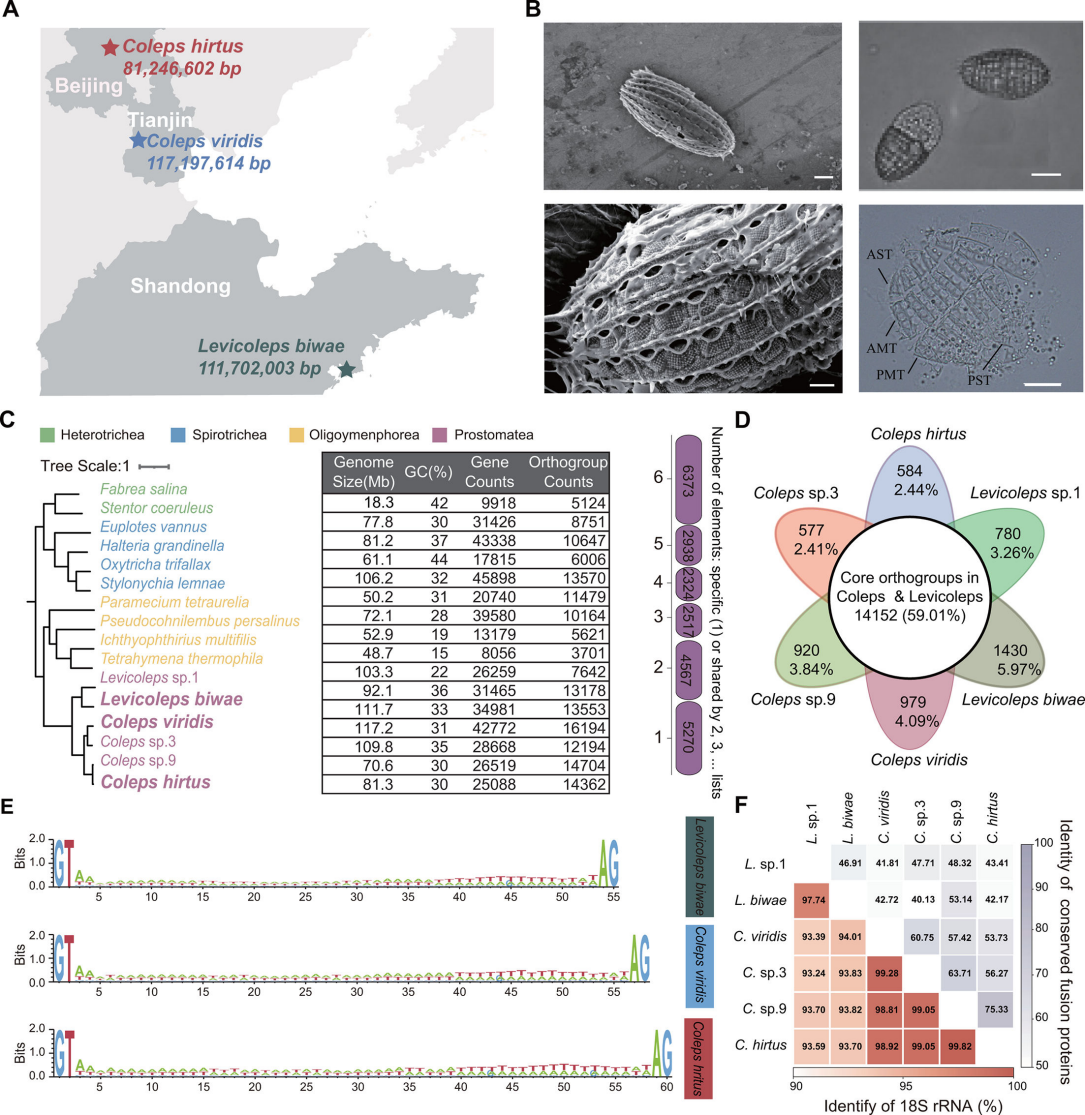
Genomic features of three *Colepidae s*pecies. (**A**) Sampling sites and genome size of *C. hirtus*, *L. biwae*, *and C. viridis*. (**B**) Scanning electron microscopy images: the whole-shell view, scale bars: 5 μm (top left); magnified shell detail, scale bars: 2 μm (bottom left). Optical microscopy images: complete and half-shell, scale bars: 10 μm (top right). Fine structure of the armor: AMT, anterior main tier; AST, anterior secondary tier; PMT, posterior main tier; PST, posterior secondary tier. Scale bars: 10 μm (bottom right). (**C**) A phylogenetic tree based on the 18S rRNA gene of 16 ciliate species; the right panel displays their respective genomic features (genome size, GC, gene counts, and orthogroup counts). (**D**) A Venn diagram showing the number of ciliate core orthogroups (CCO) present in at least three species. The values within each petal indicate the gene count and proportion for each species within the CCO, while the left panel presents a coordinate plot of repartitioned intersection sizes. (**E**) WebLogo plot of introns in three *Colepidae* species. (**F**) The bottom right displays a heatmap of the conservation of 18S sequences among different species within the *Colepidae* family, and the top right shows a heatmap of the correlation among shared genes across these species.

We collected three species of *Colepidae* ciliates from freshwater environments in Beijing, Tianjin, and Qingdao, China (Fig. 1A). Based on morphological characteristics and 18S rRNA gene sequences, these species were classified as *Coleps hirtus*, *Levicoleps biwae*, and *Coleps viridis*, all belonging to the family *Colepidae*. All three species were found to possess armored calcified plates, although they exhibited slight variations in armored morphological characteristics (15, 16). Among them, *C. hirtus* was successfully maintained in stable laboratory culture. Scanning electron microscopy (SEM) characterization revealed a mesh-like structure with small perforations on its armor (Fig. 1B). Optical microscopy (OM) further showed that the armor was composed of six tiers of rectangular plates (the circumoral, anterior secondary, anterior main, posterior main, posterior secondary, and caudal), although the circumoral and caudal tiers are barely recognizable *in vivo* (Fig. 1B). In laboratory culture, we observed binary fission in *C. hirtus*, during which two distinct armor states were apparent, namely a semi-armored state that matured into a fully armored state within 2–3 h after division.

To investigate the genetic basis of armor formation, we generated about 6.2–7.0 Gb of Illumina paired-end data for each species. After *de novo* assembly and removal of potential contaminants, the final genome sizes ranged from 81.25 to 117.20 Mb (Fig. S1). The genome quality was assessed with EUKCC (94.81%–98.70% completeness), indicating that the three genomes are of high integrity, comparable to those of the published ciliate model species (Table S1). A total of 99,325 genes were predicted using the MAKER pipeline, which integrated transcriptome data, protein homology, and *ab initio* predictions to generate high-confidence gene models (Fig. 1C). Gene length distribution exhibited a pronounced peak around 1,000 bp in all three *Colepidae* species (Fig. S2). Intron length distribution showed a major peak between 55 and 60 bp in *Colepidae*, consistent with the canonical “GT-AG” splice motif observed in other ciliates (Fig. 1E). In contrast, the conserved branch-point adenine, which is essential for intron splicing and has been previously reported near the 3′ end of introns in *Euplotes vannus* (*Spirotrichea*) (19), *Stentor coeruleus* (20), and *Fabrea salina* (*Heterotrichi*a) (21), was not detected in *Colepidae*. The absence of this branch-point adenine may indicate an accelerated mode of intron evolution, a pattern also observed in *Pseudourostyla cristata* (22), further supporting the uniqueness of this molecular characteristic.

To resolve the phylogenetic position of *Colepidae* and assess their genomic cohesion, we performed a comparative genomics analysis of 16 ciliate genomes, including 10 previously published representative ciliates and six *Colepidae* species (three from the P10K database [23] and three newly sequenced). This maximum-likelihood phylogeny revealed *Colepidae* as a well-supported monophyletic clade (Fig. 1C). OrthoFinder analysis identified 14,152 core gene families, accounting for 59.01% of all orthogroups shared among three or more *Colepidae* species. Non-core gene families, in contrast, represented only 2.41%–5.97% of the orthogroups, indicating the high degree of functional cohesion within the family (Fig. 1D).

Further comparisons of genetic markers and protein sequences reinforced our findings. For instance, 18S rRNA genes showed high identity among (98.81%–99.82% within genus *Coleps*), while *Colepidae*-specific fusion proteins clustered tightly within the genus, with identities ranging from 53.73% to 75.33% (Fig. 1F). These findings suggested that *Colepidae* has been shaped less by broad genomic divergence and more by a limited set of lineage-specific innovations. This offers a unique opportunity to pinpoint the molecular basis of their distinctive calcified coverings.

### Comparative genomics and functional analysis of genomic adaptations in *Colepidae* for biomineralization

**Fig 2:**
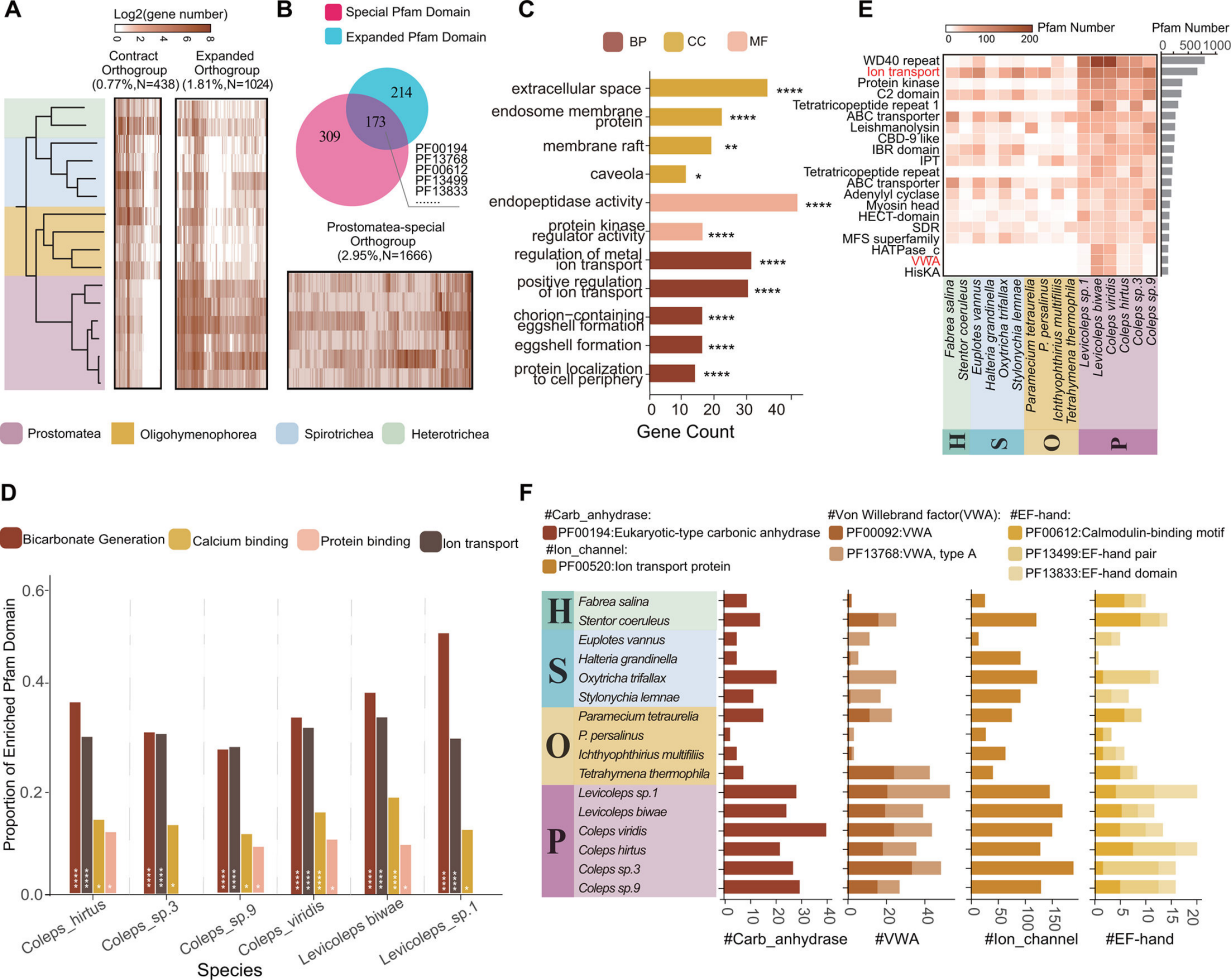
Genome and functional characterization of *Colepidae* species. (**A**) The left phylogenetic tree was constructed using 16 ciliate genomes. The right heatmap visualizes the size distribution of three gene family categories (expanded, conserved, and lineage-specific) across species, with color intensity indicating log-transformed gene counts. (**B**) A Venn diagram illustrates the overlap of functional domains in *Coleps*-expanded gene families and *Coleps*-specific gene families. (**C**) Gene Ontology (GO) enrichment analysis shows biological processes, molecular functions, and cellular components over-represented in *Colepidae*-specific genes. (**D**) Over-representation analysis identifies significantly enriched domains in *Colepidae*-specific gene families associated with calcification pathways. (**E**) A combined heatmap and bar chart displays the top 20 most abundant functional domains in expanded gene families across ciliate lineages. (**F**) A bar plot visualizes the copy number of domains involved in carbonic anhydrase activity, protein binding, calcium binding, and ion transport within expanded gene families of *Colepidae* and other ciliates. ns denotes not significant (*P* > 0.05); * denotes *P* < 0.05; ** denotes *P* < 0.01; *** denotes *P* < 0.001; and **** denotes *P* < 0.0001.

To investigate genomic adaptations in *Colepidae*, we analyzed gene family expansion and contraction across 16 ciliate genomes comprising 482,183 genes. Orthologous clustering identified 1,666 *Colepidae*-specific orthogroups (OGs). Using a birth-death model in CAFE, we revealed a significant imbalance in gene family turnover, characterized by 1,024 significantly expanded families and 438 contracted families (*P* < 0.05) (Fig. 2A; Fig. S4). Notably, 173 functional domains overlapped between *Colepidae*-specific and expanded OGs, including PF00194 (carbonic anhydrase) and PF13768 (von Willebrand factor type A domain, VWA), both critical for calcium metabolism and extracellular matrix organization (Fig. 2B).

Functional enrichment of *Colepidae*-specific families (27,167 genes) highlighted pathways linked to vesicle-mediated substrate transport (GO:0,005,901, FDR < 0.01) and extracellular ion regulation (GO:0,010,959, FDR < 0.001), consistent with the strategy of secreting calcification precursors to the cell surface (17) (Fig. 2C). Because protein domains are the fundamental units of function, we next examined which domains were enriched in calcification-related categories. Hypergeometric testing (*P* < 0.05, FDR < 0.01) identified 80.29% (*n* = 387) domains significantly associated with calcification, clustering into four major categories: bicarbonate generation, calcium binding, protein binding, and ion transport (Fig. 2D).

We then ranked domain counts within the expanded gene families. (Fig. 2E). Among the top 20 most expanded domains, we found an enrichment of calcification-related functions. These included transport-related domains (e.g., PF00005, involved in ion transmembrane transport) and protein-binding domains (e.g., PF00092, mediating protein-protein interactions). To further validate these findings, we examined four representative domain families (carbonic anhydrase, VWA, ion channel, and EF-hand), all of which showed clear expansion in *Colepidae* (Fig. 2F).

### Expansion and functional diversification of carbonic anhydrase genes in *Colepidae*

**Fig 3:**
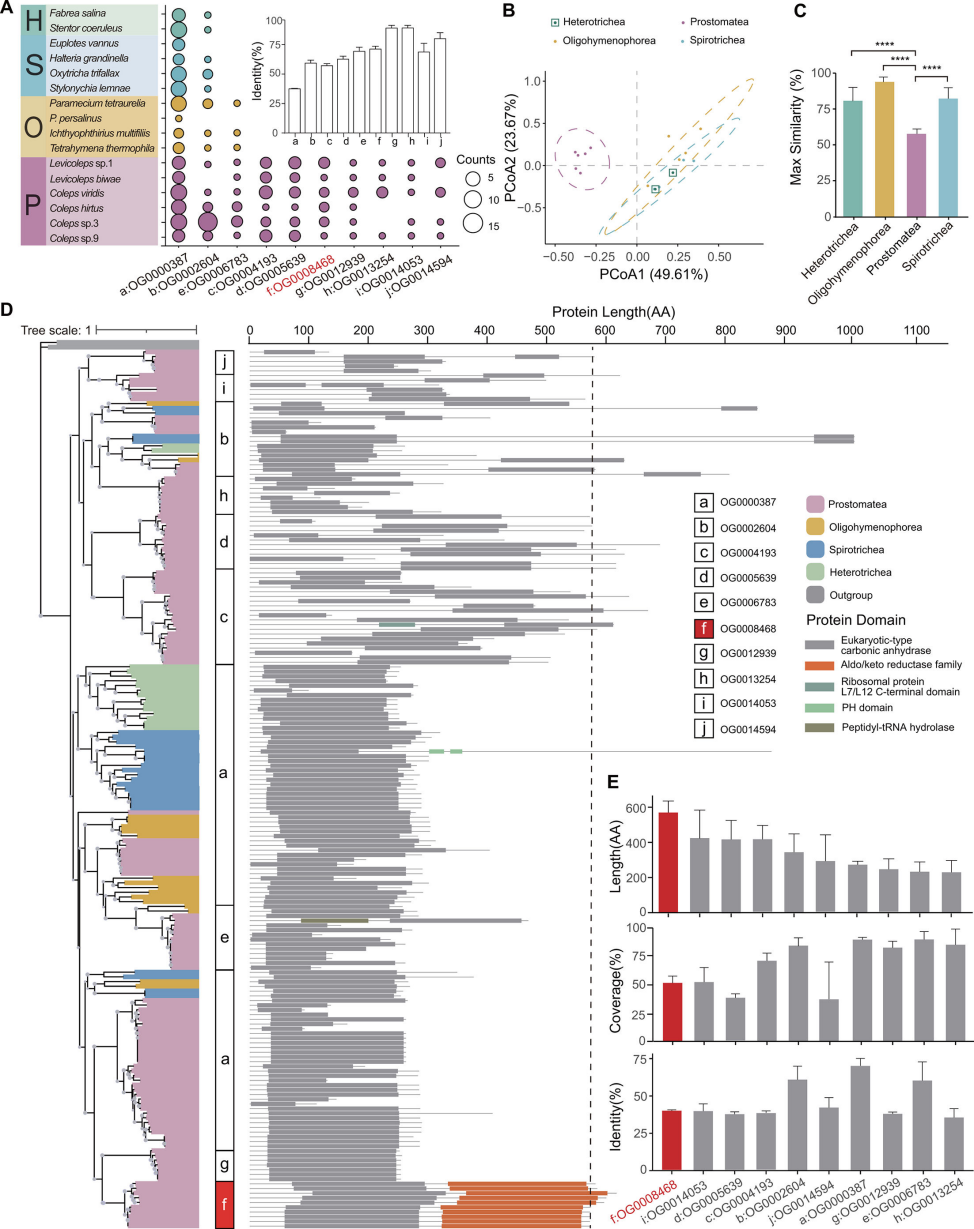
Analysis of the carbonic anhydrase family in the *Colepidae f*amily. (**A**) Distribution of the number of carbonic anhydrase genes and within-group similarity (right box plot) across 16 ciliate species. (**B**) Principal coordinate analysis (PCoA) based on variations in the number of carbonic anhydrase genes across ciliates, highlighting intergroup differences in related distributions among ciliate groups. (**C**) Comparison of maximum sequence similarity between ciliate carbonic anhydrase genes and proteins in the NR database, measured via the BLAST method. ****, *P* < 0.0001. (**D**) The left panel presents a phylogenetic tree inferred from all carbonic anhydrases. The right schematic visualizes details of protein domains (including types, boundaries) and protein lengths for all carbonic anhydrases. (**E**) Bar plot displaying corresponding protein lengths and BLAST comparison metrics (query coverage and identity) for carbonic anhydrase proteins.

Biomineralization is known to rely on the critical role of carbonic anhydrase (CA), which catalyzes the rate-limiting step of bicarbonate ion production (24, 25). In our data set of 16 ciliate genomes, we identified 254 carbonic anhydrase genes, with 179 found in the *Colepidae*. This represents a striking 3- to 5-fold expansion relative to other ciliate lineages (Fig. 3A), with principal component analysis of CA gene counts further revealing clear separation between *Colepidae* and other ciliates (Fig. 3B), indicating that lineage-specific expansion has generated a unique genomic signature. Meanwhile, OrthoFinder clustered all identified CAs into 10 orthogroups, seven of which were restricted to *Colepidae*. This finding is further corroborated by sequence similarity analysis.

Furthermore, similarity searches against the NR database revealed that *Colepidae* genes showed the lowest homology to known sequences (*P* < 0.0001) (Fig. 3C). To explore their evolutionary relationships, we reconstructed a phylogenetic tree from the catalytic *Carb* domains of all 254 genes (Fig. 3D). This analysis revealed that ciliates retain several ancestral families (e.g. OG000387 and OG0002604), accounting for approximately half of the total genes, whereas the *Colepidae*-specific carbonic anhydrases are grouped into three distinct classes (OG0008468/OG0012939, OG0005639/OG0004193, and OG0014053/OG0014594). These classes likely reflect independent evolutionary origins and functional diversification.

Within *Colepidae*, we identified a distinctive dual-domain carbonic anhydrase that combines the *Carb* domain and the *Aldo* domain (aldo-keto reductase, PF00274) (Fig. 3D). This fusion results in proteins with an average length of 570 amino acids, significantly longer than the 340-amino-acid average of other carbonic anhydrases. These dual-domain proteins are highly conserved within *Colepidae* but show limited similarity to homologs outside the group, suggesting a lineage-specific innovation (Fig. 3E).

### Evolutionary origins of the unique *Carb::Aldo* fusion protein in *Colepidae*

**Fig 4:**
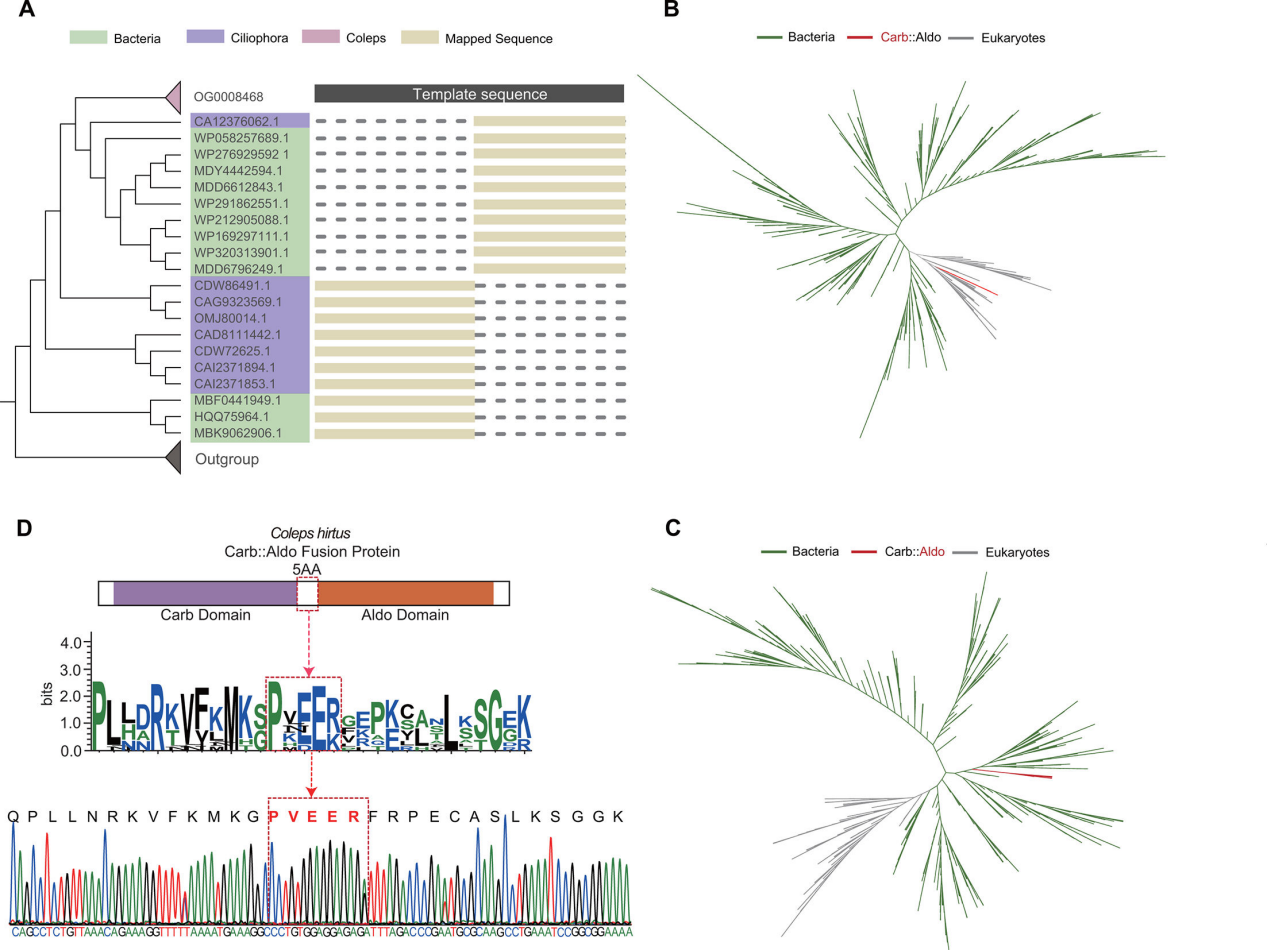
Structural and evolutionary characterization of *Carb::Aldo* fusion proteins. (**A**) Schematic diagram depicting the sequence/structural comparison between the *Carb::Aldo* fusion protein and entries in the NR database, highlighting key domains or regions of homology and divergence. (**B**) Phylogenetic tree constructed from the amino acid sequences of the *Carb* domain within the *Carb::Aldo* fusion protein, illustrating evolutionary relationships with homologous *Carb* domains from other species or proteins. (**C**) Phylogenetic tree of the *Aldo* domain portion of the *Carb::Aldo* fusion protein, depicting its evolutionary proximity to related *Aldo* domains based on sequence similarity. (**D**) Panel showing the amino acid composition of the linker region in the *Carb::Aldo* fusion protein (top) alongside Sanger sequencing results (bottom), verifying the nucleotide sequence corresponding to the Linker region.

To investigate the evolutionary origins of the *Carb::Aldo* fusion protein, we searched both the NR and P10K databases. No full-length proteins containing both domains were detected outside *Colepidae* (Fig. 4A). In addition, motif analysis and alignment results for similar proteins also showed similar results (Fig. S5 and S6). These results indicate that the fusion protein reported here represents a previously undescribed domain combination restricted to *Colepidae*, highlighting a lineage-specific structural innovation potentially linked to their biomineralization strategy.

Phylogenetic analyses of individual domains supported distinct evolutionary origins. The *Carb* domain clustered with homologs from eukaryotic ciliates, consistent with vertical inheritance (Fig. 4B). In contrast, the *Aldo* domain was observed to group with bacterial *aldo-keto reductases* (Fig. 4C). The placement of the *Colepidae Aldo* domain within a bacterial clade suggests a potential prokaryotic affiliation, raising the possibility that this module might have been acquired through horizontal gene transfer. Given the metabolic roles of bacterial *aldo-keto reductases* in redox reactions and carbohydrate metabolism, the acquisition of this domain may have provided *Colepidae* with enhanced capabilities for energy balance or local pH regulation—processes essential for sustaining calcification.

Structural modeling with AlphaFold3 predicted a compact conformation in which the two domains are linked by a five-residue flexible linker (Fig. 4D; Fig. S7). This arrangement places the bicarbonate-generating *Carb* catalytic core in proximity to the *Aldo* active site, suggesting a potential functional coupling of redox and catalytic reactions. Such a configuration may allow the *Aldo* domain to channel NAD(P)H-dependent reducing power or modulate glycolytic flux, thereby supporting the *Carb* domain in supplying bicarbonate for calcium carbonate deposition (26). Genomic confirmation of the fusion, including conserved linker sequences and syntenic context in *C. hirtus*, further supports its stable integration and adaptive retention within the *Colepidae* lineage.

### Transcriptomic analysis and RT-qPCR validation of armor regeneration

**Fig 5:**
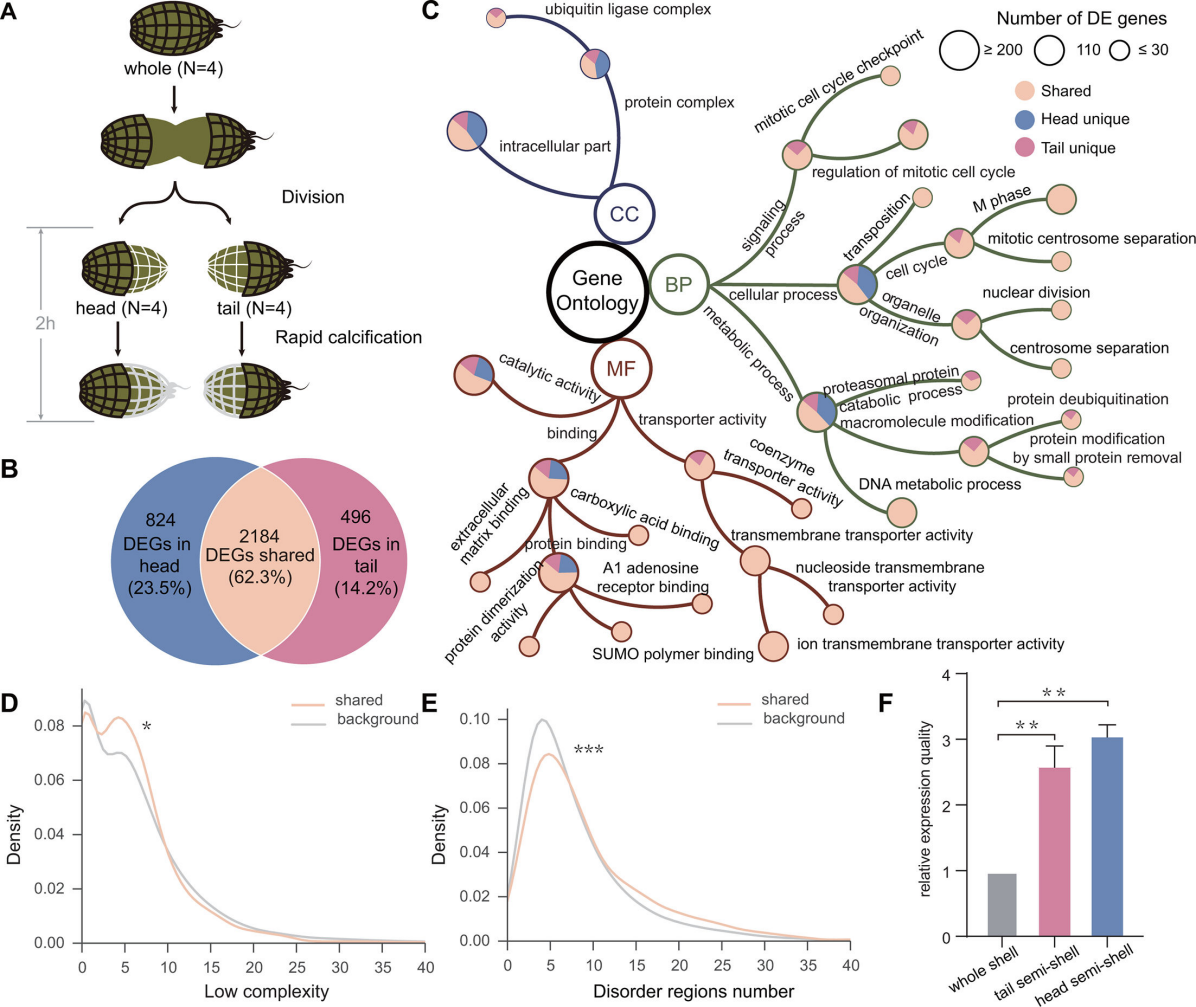
Transcriptomic analysis and RT-qPCR validation of shell regeneration. (**A**) Categorized *C. hirtus* into three groups based on shell morphology: whole armor, head semi-armor, and tail semi-armor. Four samples were collected from each group for transcriptomic analysis. (**B**) Venn diagram of differentially expressed genes in the two groups. (**C**) GO and KEGG enrichment analysis of DEGs in different categories. The color of the dots represents the *P* value. (**D and E**) Density plots of low-complexity region (LCR) proportion and number of intrinsically disordered regions (IDRs) for shared differentially expressed genes are shown in red, while the gray background represents the distribution of the entire *C. hirtus* proteome (LCR *P* = 0.0101, IDR *P* = 6.5592e-19), based on Wilcoxon rank-sum test. (**F**) Quantitative real-time PCR analysis of Carb::Aldo fusion gene expression levels in different groups. ns denotes not significant (*P* > 0.05); * denotes *P* < 0.05; ** denotes *P* < 0.01; and *** denotes *P* < 0.001.

To examine how genomic changes translate into functional regulation, we analyzed the transcriptomic dynamics during rapid armor regeneration in *C. hirtus*. Cells were categorized into three morphological states (whole armor, head semi-armor, and tail semi-armor) to capture different stages of calcification (Fig. 5A). This framework enabled us to identify the dynamic transcriptional programs associated with shell repair following division.

Differential expression analysis revealed 3,008 genes differing between the head and whole-armor groups, and 2,680 genes between the tail semi-armor and whole-armor groups. Of these, 2,184 (62.3%) were shared, while 824 (23.5%) and 496 (14.2%) were specific to the head and tail, respectively (Fig. 5B; Data S1). Functional enrichment of the shared set highlighted processes central to regeneration, including exocytosis, phosphorylation, and regulation of endocytosis, as well as enzyme binding and ion channel regulation. KEGG analysis further emphasized pathways such as calcium signaling and focal adhesion, consistent with coordinated cellular activities during regeneration (Fig. S8). Together, these results emphasize a complex regulatory network that supports efficient armor formation (Fig. 5C; Data S2).

Because biomineralization in many lineages involves proteins with low complexity and intrinsic disorder, which act as flexible templates for crystal nucleation (27), we examined the sequences of the shared differentially expressed genes. These proteins exhibited unusually low sequence complexity and a strong propensity for disorder. This is consistent with previous findings in the calcification mechanisms of coccolithophores (27) (Fig. 5D and E). Notably, RT-qPCR analysis of *Carb::Aldo* revealed significantly increased expression in the head and tail regions of the half shell compared to the whole shell (*P* = 0.0039 and *P* = 0.0011, respectively) based on one-way ANOVA, suggesting its direct involvement in the rapid calcification process (Fig. 5F).

### RNAi-driven knockdown of *Carb::Aldo* fusion protein disrupts calcified armor formation

**Fig 6:**
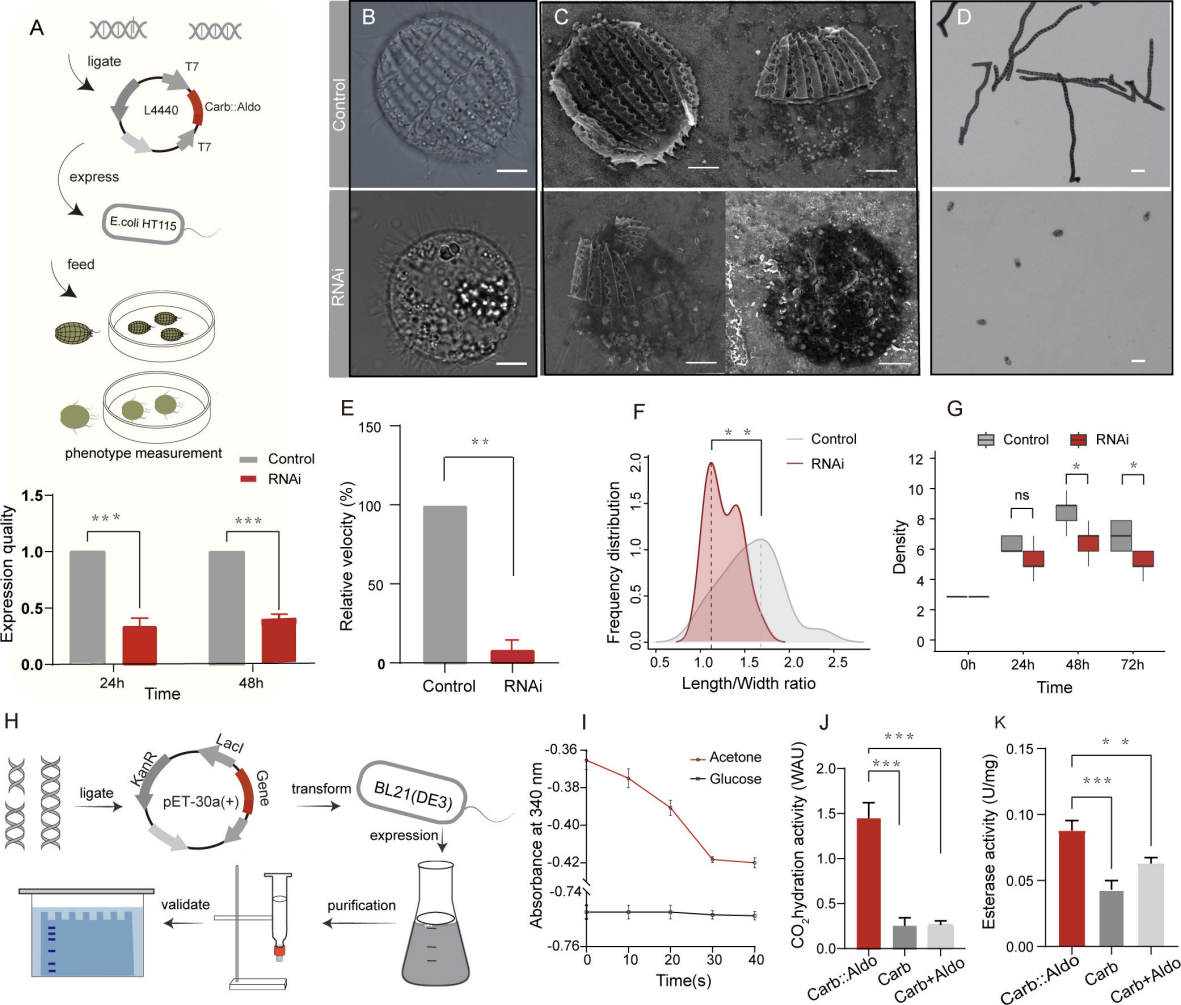
Functional characterization of the *Carb::Aldo* fusion gene via RNAi-mediated knockdown and *in vitro* biochemical assays. (**A**) Schematic illustration of the RNAi experimental procedure. The *Carb::Aldo* fusion gene sequence was cloned into the L4440 plasmid, expressed in *E. coli* HT115, and then fed to *C. hirtus* to induce gene silencing. The phenotype was measured after the cells were fed with *E. coli* HT115. The bar graph shows the gene expression after RNAi using 18S rRNA as an internal reaction control. (**B and C**) Optical and electron microscopy images showing the calcified armor phenotypes of *C. hirtus*, respectively. Control cells display normal whole and semi-armor structures, while RNAi-treated cells show partial or complete armor loss; scale bar: 10 μm. (**D and E**) Results of locomotion assays. The top panel (**D**) shows representative movement trajectories of control and RNAi-treated cells, and the bottom panel (**E**) presents the relative velocity of the two groups. (**F**) Frequency distribution of the length/width ratio of control and RNAi-treated group (*n* = 18 cells for each group). (**G**) Growth analysis of control and RNAi-treated cells at 0, 24, 48, and 72 h post-RNAi treatment (*n* = 5). (**H**) Schematic workflow for heterologous expression and purification. (**I**) Catalytic activity of *Aldo* protein toward acetone and glucose. (**J**) CO₂ hydration activity of carbonic anhydrase (based on one-way ANOVA, both *P* < 0.0001). (**K**) Esterase activity of carbonic anhydrase (based on one-way ANOVA, *P* = 0.0003 and *P* = 0.0061, respectively). ns denotes not significant (*P* > 0.05); * denotes *P* < 0.05; ** denotes *P* < 0.01; and *** denotes *P* < 0.001.

To test the functional significance of the *Carb::Aldo* fusion gene, we applied RNA interference (RNAi) (28) in *C. hirtus*. Feeding assays successfully reduced *Carb::Ald*o transcript levels, with qPCR confirming significant downregulation at 24 and 48 h (both group *P* < 0.001, based on two-way ANOVA) (Fig. 6A). Silenced cells displayed marked structural defects, including increased transparency, loss of ridge-like surface features, and partial or complete detachment of calcified plates (Fig. 6B and C). These morphological alterations were accompanied by impaired motility: RNAi-treated cells swam more slowly and followed restricted trajectories relative to controls (*P* = 0.0019, based on unpaired t test with Welch’s correction) (Fig. 6D and E). Cell shape shifted from the characteristic barrel form to a pea-like morphology, with a significant reduction in the length-to-width ratio (1.68 vs 1.12, *t*-test, *P* = 0.0022) (Fig. 6F; Fig. S9A; Data S3).

RNAi treatment also inhibited cell proliferation, as shown by growth curve analyses at 24, 48, and 72 h (*P* = 0.1163, *P* = 0.0133, and *P* = 0.0133, respectively, based on two-way ANOVA) (Fig. 6G). The loss of armor further reduced intraspecific competitiveness: unprotected cells were surrounded and preyed upon by conspecifics (Fig. S9B). Together, these results demonstrate that *Carb::Aldo* is indispensable for calcified armor formation, cell morphology, motility, and fitness, underscoring its central role in the physiology of *C. hirtus*.

### Functional synergy within the *Carb::Aldo* fusion architecture

To determine whether the physical linkage of the *Carb* and *Aldo* domains confers a catalytic advantage, we heterologously expressed and purified *Carb::Aldo* fusion protein, the isolated *Carb* domain, and the *Aldo* domain from *E. coli*. SDS-PAGE and Western blot analysis confirmed successful purification of all three proteins, with observed molecular weights consistent with predictions (Carb::Aldo ~70 kDa, Carb ~26.9 kDa, Aldo ~31.1 kDa) (Fig. 6H; Fig. S9C and D).

We first assessed the catalytic viability of the HGT-derived modules. The isolated *Aldo* domain exhibited detectable NADPH-dependent reductase activity toward acetone (0.016 U/mg), a standard model substrate, while no activity was detected toward glucose (Fig. 6I). This result reveals that the *Aldo* domain is a structurally intact and catalytically viable module rather than a degenerate pseudogene. Next, we evaluated whether the fusion architecture enhances the activity of the Carb domain. The CO₂ hydration assay revealed that the *Carb::Aldo* fusion protein exhibited a specific activity of 28.4 WAU/mg, which was significantly higher than that of the Carb domain alone (5.2 WAU/mg) or a mixture of *Carb* and *Aldo* (5.4 WAU/mg) (both *P* < 0.0001, based on one-way ANOVA) (Fig. 6J). This synergistic effect was further corroborated by esterase activity assays, where the fusion protein (0.078 U/mg) outperformed the isolated *Carb* domain (0.043 U/mg) or a mixture of *Carb* and *Aldo* (0.063 U/mg) (*P* = 0.0003 and *P* = 0.0061, respectively, based on one-way ANOVA) (Fig. 6K). These *in vitro* results demonstrate that the fusion of the two domains enhances the catalytic performance of the *Carb* domain, providing direct evidence for functional synergy within the *Carb::Aldo* architecture.

## DISCUSSION

Unlike most ciliates that rely on pellicles (29) or temporary shells (30), members of *Colepidae* achieve long-term protection and ecological adaptation through the formation of highly ordered calcium carbonate shells (17). This mineralization capability enables them to occupy unique ecological niches in freshwater and brackish ecosystems. In this study, we provide a holistic view of biomineralization mechanisms in ciliates by integrating high-quality genomes of *Colepidae* species, conducting robust phylogenetic, functional, and transcriptomic analyses. By linking genomic innovations (such as gene family expansions and domain fusions) to phenotypic adaptations like calcified shell formation, our multi-omics approach bridges critical gaps in the understanding of biomineralization processes.

While biomineralization occurs across diverse lineages (from mollusks forming shell matrices (31) to diatoms with siliceous walls (32) and coccolithophores producing calcareous scales (33), the *Colepidae* employ a uniquely sophisticated mineralization strategy. Unlike multicellular organisms, which rely on specialized tissues (e.g., molluscan mantle cells) (34), hormonal control (35), or unicellular algae with extracellular scale production, the *Colepidae* demonstrate precise intracellular control over calcification. Their biomineralized shells show highly species-specific structures, displaying unique plate-like morphologies (17). These structures are formed through finely regulated processes after cell division. Their rapid shell regeneration suggests the existence of an efficient and precise calcification regulatory system. However, the underlying mechanisms had remained largely uncharacterized.

Previous studies have demonstrated how the genomes of calcifying organisms are adapted to support biomineralization processes (31–35). Our comparative genomic analysis of ciliates corroborates these findings and reveals unique mechanisms underlying the biomineralization of *Colepidae*. Compared to other ciliates, the *Colepidae* genomes show significant expansions in gene families associated with carbonic anhydrases, calcium binding, protein binding, and ion transport.

A total of 173 functional domains are shared in *Colepidae*-specific and expanded orthogroups, linking them closely to calcium metabolism and extracellular matrix formation. Transcriptomic analysis during shell regeneration demonstrates coordinated upregulation of genes involved in calcium signaling, vesicle trafficking, and extracellular matrix remodeling. The enrichment of low-complexity and intrinsically disordered proteins among differentially expressed genes mirrors mechanisms observed in other biomineralizing organisms (e.g., coccolithophores), in which disordered regions facilitate mineral nucleation (27). Collectively, these findings indicate that genomic adaptations in *Colepidae* underpin their efficient biomineralization.

Studies on biomineralization in protists have highlighted the importance of carbonic anhydrase genes in calcification (24). The expansion of carbonic anhydrase genes in *Colepidae* far exceeds that in other ciliates, aligning with their reliance on rapid bicarbonate production for calcium carbonate deposition. This expansion is not merely quantitative but also reflects a high degree of functional diversification, as evidenced by the emergence of *Colepidae*-specific carbonic anhydrase families. Phylogenetic divergence of these carbonic anhydrase families from ancestral forms suggests adaptive evolution tailored to optimize calcification efficiency. Significant sequence differences between *Colepidae* carbonic anhydrase genes and their homologs in other eukaryotes further support their specialized role in biomineralization. Such genomic innovations likely enable *Colepidae* to construct complex shell structures.

Another intriguing discovery in this study is the identification of the *Carb::Aldo* fusion protein, which combines a carbonic anhydrase domain with a bacterial-derived *aldo-keto reductase* domain. Structural modeling of this fusion protein predicts a functional synergy between the proximity of *Carb* and *Aldo* active sites: the *Carb* domain catalyzes bicarbonate production, while the *Aldo* domain may regulate local pH or energy flux via NAD(P)H-dependent reactions (26). This fusion represents a novel evolutionary strategy that recruits bacterial metabolic modules for biomineralization, thereby expanding the functional repertoire of ciliate genomes. The conserved homology and dynamic expression of this fusion gene across *Colepidae* species suggest its adaptive significance in shell formation. Our experiments targeting the *Carb::Aldo* fusion gene during regeneration underscore its critical role in coupling metabolic activity with calcification. These findings highlight the importance of the *Carb::Aldo* fusion protein in post-division survival and the essential process of shell regeneration.

Extensive studies have demonstrated that horizontal gene transfer (HGT) is a key driver of functional diversity in eukaryotes, particularly in acquiring metabolic and stress-adaptation genes (36). Our phylogenetic analyses suggest that the *Aldo* domain originated from an ancient bacterial HGT event. While the dynamic turnover of ciliate symbioses over deep evolutionary time precludes identification of the original donor (37), the functional implications of this transfer are profound. The *Carb::Aldo* fusion represents a modular genomic innovation. By physically tethering bacterial redox regulation (*Aldo*) to eukaryotic bicarbonate synthesis (*Carb*), the host ciliate successfully co-opted a prokaryotic metabolic module to optimize eukaryotic biomineralization. This functional coupling likely enabled the organism to better modulate local chemical gradients, providing a tangible adaptive advantage in environments with fluctuating pH. This modular evolutionary strategy circumvents the high cost of *de novo* evolution and facilitates adaptation to diverse environments (38).

In summary, this study elucidates the genomic adaptive evolution and molecular basis of biomineralization in *Colepidae* ciliates. The expansion of carbonic anhydrase genes and the emergence of the *Carb::Aldo* fusion protein via HGT illustrate the diverse mechanisms driving ecological specialization in protists. Our findings deepen our understanding of protists and provide broader insights into the evolution of biomineralization, a process with profound ecological and biogeochemical implications. Future work should explore the functional roles of *Colepidae*-specific genes through targeted mutagenesis and assess the ecological consequences of their calcification strategies in natural environments. These efforts will further bridge the gap between genomic innovations and organismal adaptations in microbial eukaryotes.

## MATERIALS AND METHODS

### Cell isolation, culture, morphological analysis, and species identification

Three samples (*C. hirtus*, *L. biwae*, *C. viridis*) were collected from freshwater environments in Beijing (116°30.45′E, 40°20.15′N), Tianjin (116°58.50′E, 39°09.69′N), and Qingdao (120°20.78′E, 36°04.10′N), China. Single cells were isolated using a glass micropipette under a stereomicroscope. Based on morphological characteristics and 18S rRNA gene sequencing, these species were classified into three distinct taxa, representing two major genera (*Coleps* and *Levicoleps*) within the family *Colepidae*.

### Genome assembly

FastQC v0.12.1 was used to evaluate sequencing data from the same species, which was then merged. Adapter trimming and quality filtering were carried out using Fastp v0.23.4 (39) and Trimmomatic v0.39 (40) (parameters: LEADING:20, TRAILING:20, SLIDINGWINDOW:4:25, MINLEN:120, AVGQUAL:28). *De novo* genome assembly for the three *Colepidae* species was conducted using SPAdes v3.13.0 (41) with single-cell and multiple k-mer options (--sc -m 500 -t 50 -k 61,71,77,81), followed by filtering out contigs shorter than 1 kb. To remove potential contamination, the following steps were applied:

Kraken2 v2.1.3 (42) (--db database_Nr --confidence 0.1) was used to identify contaminant sequences by comparing the genome draft to the Nr database. Sequences assigned to bacterial or algal taxa were considered contaminants. Highly homologous sequences were removed using CD-HIT v4.8.1 (43) (cd-hit-est -c 0.95 -n 10 -d 0 -M 16,000), and Purge_dups v1.2.5 (44) was applied to eliminate haplotypic and redundant sequences based on read depth. Genome assembly statistics were assessed using QUAST v5.2.0 (45), while genome completeness was evaluated at the protein level using EukCC v2.1.0 (46). BUSCO v5.6.1 was used to assess genome quality based on the Alveolata data set in protein mode.

### Gene prediction

To characterize genetic code usage in ciliates, gene prediction and annotation were performed using Codetta v2.0 (47, 48). Repetitive elements were masked with RepeatMasker v4.1.3 (49), and a *de novo* repeat library was constructed using RepeatModeler to minimize false-positive predictions. Gene prediction was conducted with MAKER v3.01.04 (50), incorporating transcriptome data generated in this study. The data set included 23 Gb of paired-end 150 bp (PE150) RNA-seq reads, which underwent adapter removal and quality filtering. *De novo* transcriptome assembly was performed using Trinity v2.11.0 with default parameters, followed by redundancy removal with CD-HIT v4.8.1 at a 95% similarity threshold.

To enhance prediction accuracy, amino acid sequences from 10 published ciliate species were used as cross-species homologous protein evidence. *Ab initio* gene prediction was carried out using SNAP (51), AUGUSTUS (52), and GeneMark-ES (53), and the initial annotations were integrated into MAKER. The *ab initio* predictors (SNAP and AUGUSTUS) were further trained using preliminary MAKER annotations to refine species-specific gene models through iterative updates.

### Functional enrichment analysis

Protein-coding gene functions were inferred by identifying conserved motifs and domains with InterProScan v5.66-98.0 (54), using parameters (-iprlookup -goterms). Predicted proteins were annotated against multiple databases, including PROSITE, HAMAP, PFAM, PRINTS, PRODOM, SMART, TIGRFAMS, PIRSF, SUPERFAMILY, CATH-GENE3D, and PANTHER. Functional annotation was also performed using eggNOG-mapper (55) to assign Gene Ontology (GO) terms and KEGG pathways.

For non-coding RNA annotation, tRNAscan-SE v2.0.7 (56) was used to identify tRNA genes under default parameters. Transcriptome data were searched against the Nr database using DIAMOND v0.9.26 (57). Functional annotation data from InterProScan, eggNOG-mapper, and DIAMOND were merged to ensure maximum accuracy, retaining genes identified by at least one of the three tools.

To further explore the biological significance of annotated genes, functional enrichment analysis was conducted. KEGG orthology (KO) terms were assigned using KofamScan v1.3.0 (58) (E-value ≤ 0.05). Functional enrichment analysis was performed using the enricher function from the R package clusterProfiler v3.12.0 (59).

### Orthologous group analysis

We constructed genome-wide orthologous groups (OGs) using OrthoFinder v2.5.5 (60). First, we performed all-vs-all protein sequence similarity searches with DIAMOND v2.0.15.153, using a hypergeometric distribution-optimized alignment strategy (--more-sensitive --evalue 1e-5). For gene pairs with sequence similarity above the threshold, we conducted multiple sequence alignment with MAFFT (61) and built gene family phylogenetic trees using FastTree (62) (-gamma -lg). Finally, we clustered protein sequences from 16 species into OGs using the Markov clustering algorithm and calculated the matrix of co-linearity orthologous genes between species. Using OrthoFinder, we clustered a total of 482,183 genes into 56,533 orthologous groups (OGs), with 86.9% of genes assigned to OGs.

For phylogenetic tree construction, we combined concatenation and coalescence methods. We first built a concatenated sequence matrix from single-copy orthologs (SCOs) to derive a super-gene, then constructed a maximum likelihood tree with IQ-TREE v2.2.0.3 (63), using ModelFinder Plus and 1,000 bootstrap replicates. The tree topology was visualized and optimized on the iTOL (64) platform, with a focus on highlighting the phylogenetic positions of *Colepidae* species and their relationships with closely related taxa.

### Phylogenetic and gene family analysis

To systematically analyze the genomic characteristics of *Colepidae* species, we collected data from 10 model species across three different families of the ciliate class, including species such as *Tetrahymena thermophila* and *Paramecium tetraurelia*. We also incorporated genome data from three *Colepidae* species from the P10K public database and three newly sequenced *Colepidae* species from this study. In the genome homology analysis phase, we used OrthoFinder v2.5.5 to construct genome-wide orthologous groups (OGs). For specific usage, please refer to the above content.

To obtain calibration points, we used the Timetree database (65) to get the divergence time of the outgroup species *T. thermophila* and *Ichthyophthirius multifiliis* (227 million years ago). Molecular clock dating was performed using the r8s v1.81 (66) under a Bayesian framework. Further analysis of gene family expansions and contractions was carried out using CAFE v5.0 (67).

### Carbonic anhydrase gene identification

Candidate genes with the PF00194 domain were filtered via custom scripts and verified manually for conserved catalytic domains. Using orthologous groups from OrthoFinder, we extracted gene families with the PF00194 domain, selecting only those with at least two species and three proteins to ensure robust analysis. We aligned 254 carbonic anhydrase protein sequences and two external controls using MAFFT. Low-quality alignment regions were removed with TrimAl v1.4.rev15 (68), and a phylogenetic tree was built with IQ-TREE, using 1,000 bootstrap replicates to calculate branch support. Domain illustrations were created and refined using iTOL.

### Alignment of carbonic anhydrase

We performed BLASTp searches using DIAMOND on 254 candidate carbonic anhydrase genes against the NR database in sensitive mode (--sensitive) to detect distant homology, requiring identity ≥30% and coverage ≥50%. The identity distribution differences between *Coleps* and non-*Coleps* species were compared using Student’s *t*-test (significance threshold *P* < 0.01), with Benjamini-Hochberg correction for multiple testing (FDR < 0.05). Amino acid counts were tallied via SeqKit v2.3.0 (69) stats module, and sequence-to-sequence alignments were conducted using DIAMOND.

### HGT analysis of the Carb::Aldo fusion protein

To analyze the HGT events of the *Carb::Aldo* fusion protein, we used InterProScan to determine the precise boundaries of the *Carb* (PF00194) and *Aldo* (PF00274) domains. Sequences with overlaps or breaks (coverage < 90%) were excluded. DIAMOND v2.0.15.153 BLASTp was used in sensitive mode to retrieve the top 1,000 homologous sequences for each domain. Multiple sequence alignment was performed using MAFFT, followed by trimming with TrimAl and phylogenetic tree construction using IQ-TREE with 1,000 Ultrafast bootstrap replicates. HGT events were identified through manual analysis of the tree topology.

### Transcriptomic analysis

*C. hirtus* was categorized into three morphological groups: head semi-armor, tail semi-armor, and whole armor. A total of 115.4 Gb of transcriptomic data were obtained. Raw data were quality-checked and cleaned using FastQC and Fastp to remove low-quality sequences and adapter contaminants. The cleaned reads were aligned to the reference genome using STAR v2.7.11a (70). Gene expression matrices were generated via featureCounts v2.0.6 (71) based on gene annotation files. Differential expression analysis using DESeq2 (72) identified 3,504 significantly differentially expressed genes compared with the whole-shell group. GO classification was performed with BiNGO v3.0.5 (73), and *P* values were calculated using the hypergeometric test. The ProminTools suite (74) was used to evaluate the disorder and low complexity of protein sequences from shared differentially expressed genes.

### RNA interference

Total RNA of *C. hirtus* was extracted using the Trizol method to obtain high-quality total RNA. Single-stranded cDNA was synthesized using the Hifair III 1st Strand cDNA Synthesis SuperMix for qPCR, which includes a gDNA digester to minimize genomic DNA contamination. Two pairs of primers were designed. The first pair (82i2_F: CGAACAAGCACAATTAGTCA, 82i2_R: CTCTGAATGTGGTGCGTAT) was used for conventional PCR, whose product was ligated to the vector L4440 to transcribe and generate dsRNA. The second pair of primers (82q2_F: AAGGAGGAGATAAGGAGGAA, 82q2_R: GCAGATACTCGCTCTCAG) was used for qPCR to detect the expression level of Carb::Aldo. PCR products were validated using 1% agarose gel electrophoresis to confirm the correct size and purity. The purified PCR product was ligated into the pMD19-T vector and transformed into JM109 competent cells. White clones were picked for plasmid extraction. The segment ligated into the L4440 plasmid was digested using *Kpn* I and *Hind* III restriction enzymes. The resulting plasmids were transformed into *E. coli* HT115 competent cells via heat shock. Positive clones were screened using LB plates containing tetracycline and ampicillin. The L4440 plasmid carries two T7 promoters and can express double-stranded RNA (dsRNA) upon induction with 0.4 mmol/L IPTG. Bacteria cultured to the early logarithmic phase (OD_600_ = 1) were harvested, washed twice with sterile water, and then added to the RNAi experimental system containing *C. hirtus* cells, IPTG, and ampicillin. Control cells were fed HT115 cells transformed with empty L4440 plasmids. Fresh cultures of *E. coli* HT115 were prepared daily to ensure optimal dsRNA expression. The dsRNA expressed by the L4440 plasmid was released into the cytoplasm of host cells to mediate target mRNA degradation. Regular phenotypic observations were conducted to assess the RNAi effect and document phenotypic changes in *C. hirtus*.

### Heterologous expression and *in vitro* activity assays of *Carb*, *Aldo*, and *Carb::Aldo* proteins

The codon-optimized coding sequences of the *Carb::Aldo* fusion gene, the *Carb* domain alone, and the *Aldo* domain alone were synthesized and cloned into the pET-30a(+). The recombinant plasmids were transformed into *E. coli* BL21(DE3) competent cells. Protein expression was induced with IPTG at 15°C overnight. The soluble fractions were collected and subjected to Ni-NTA affinity chromatography. Purified proteins were analyzed by SDS-PAGE and Western blot using an anti-His tag antibody.

Carbonic anhydrase activity was assessed using both the CO₂ hydration assay and the esterase assay. For the CO₂ hydration assay, the reaction was performed on ice in Tris-HCl buffer containing bromothymol blue and CO₂-saturated water. The time for the solution to change from blue to yellow was recorded, and activity was expressed in Wilbur-Anderson units per milligram of protein. For the esterase assay, the hydrolysis of p-nitrophenyl acetate was monitored spectrophotometrically in Tris-SO₄ buffer. One unit of activity was defined as the formation of one micromole of p-nitrophenol per minute per milligram of protein. For the *Aldo* domain, the NADPH-dependent reductase activity was measured by monitoring the absorbance decrease at 340 nm in a reaction system containing PBS, NADPH, the specific substrate, and the purified protein.

## Data Availability

Raw sequence data, including DNA-seq (CRA028622) and RNA-seq (CRA028649), together with the assembled genomes (GWHGGYD00000000.1, GWHGGYE00000000.1, and GWHGGYF00000000.1), have been deposited at the National Genomics Data Center (NGDC) under the BioProject accession PRJCA043692.

## SUPPLEMENTAL MATERIAL

Data S1 mbio.03654-25-s0001.xlsx Source data for Fig. 5B.

Data S2 mbio.03654-25-s0002.xlsx Source data for Fig. 5C.

Data S3 mbio.03654-25-s0003.xlsx Source data for Fig. 6F.

Supplemental material mbio.03654-25-s0004.docx Figures S1-S9; Table S1.

ASM does not own the copyrights to Supplemental Material that may be linked to, or accessed through, an article. The authors have granted ASM a non-exclusive, world-wide license to publish the Supplemental Material files. Please contact the corresponding author directly for reuse.
