# Supplementary material for: Bacterial domain fusion drives biomineralization innovation in Colepidae ciliates
Source: mBio. 2026 Jun 3;17(7):e03654-25. doi: 10.1128/mbio.03654-25 (PMC13344042; doi:10.1128/mbio.03654-25)
Supplement: Supplemental material — Figures S1-S9; Table S1. [file mbio.03654-25-s0004.docx]

Supplementary Materials for

*Bacterial domain fusion drives biomineralization innovation in Colepidae ciliates*

Keke Wu^1^^ , Wenyu Chen^2^^ , Chenghu Fan^1^, Xuqi Lu^1^, Bing Zhang^3,4,*^, Miao Miao^1, *^

^ contributed equally

*Corresponding author (M.M., miaomiao@ucas.ac.cn; B.Z. BingZhang@ucas.ac.cn)

**This PDF file includes:**

Figs. S1 to S9

Tables S1

**Figure S1.**


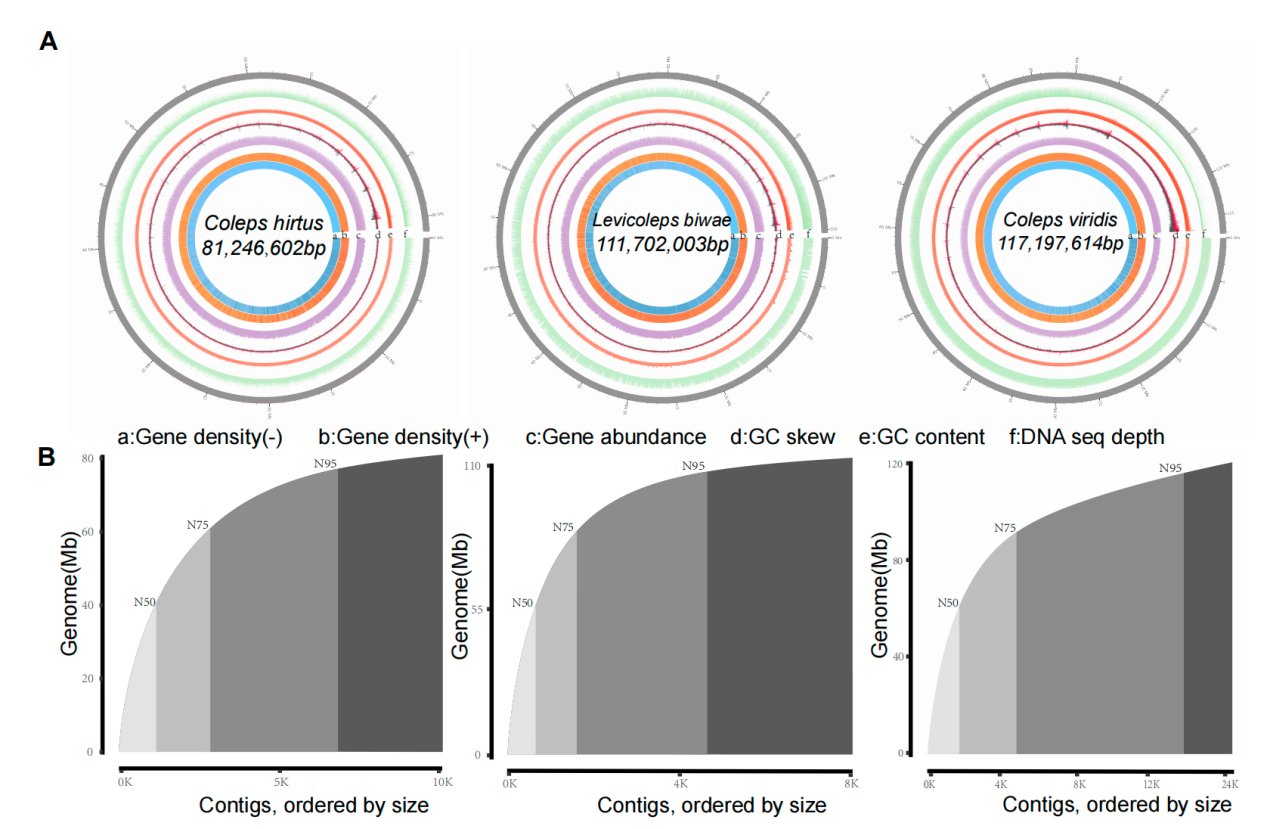


**Figure S1 Genome assembly for the Three Colepidae Species.** A Circular genome plots of the three Colepidae species: *C. hirtus*, *L. biwae*, and *C. viridis*. Each circle represents different genomic features, including gene density, Gene abundance, GC skew, GC content, and DNA seq depth. B Genome assembly metrics for the three Colepidae species. Each panel displays the distribution of contigs ordered by size, with the N50, N75, and N95 values indicated.

**Figure S2.**


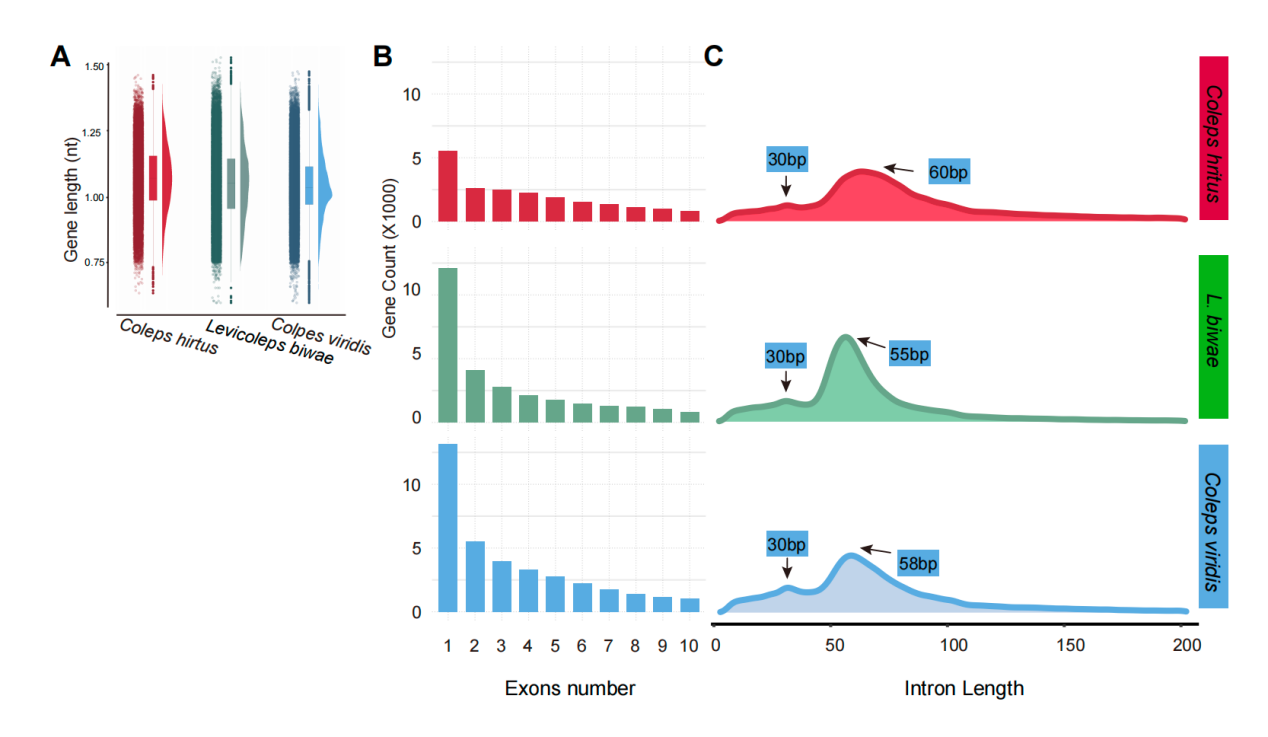


**Figure S2 Genomic Characteristics of Three Colepidae Species.** A showing the distribution of gene lengths in the three Colepidae species. B Histograms displaying the gene count distribution across different exon numbers. C Intron length distributions for the three species, with peaks indicating the most frequent intron lengths.

**Figure S3.**


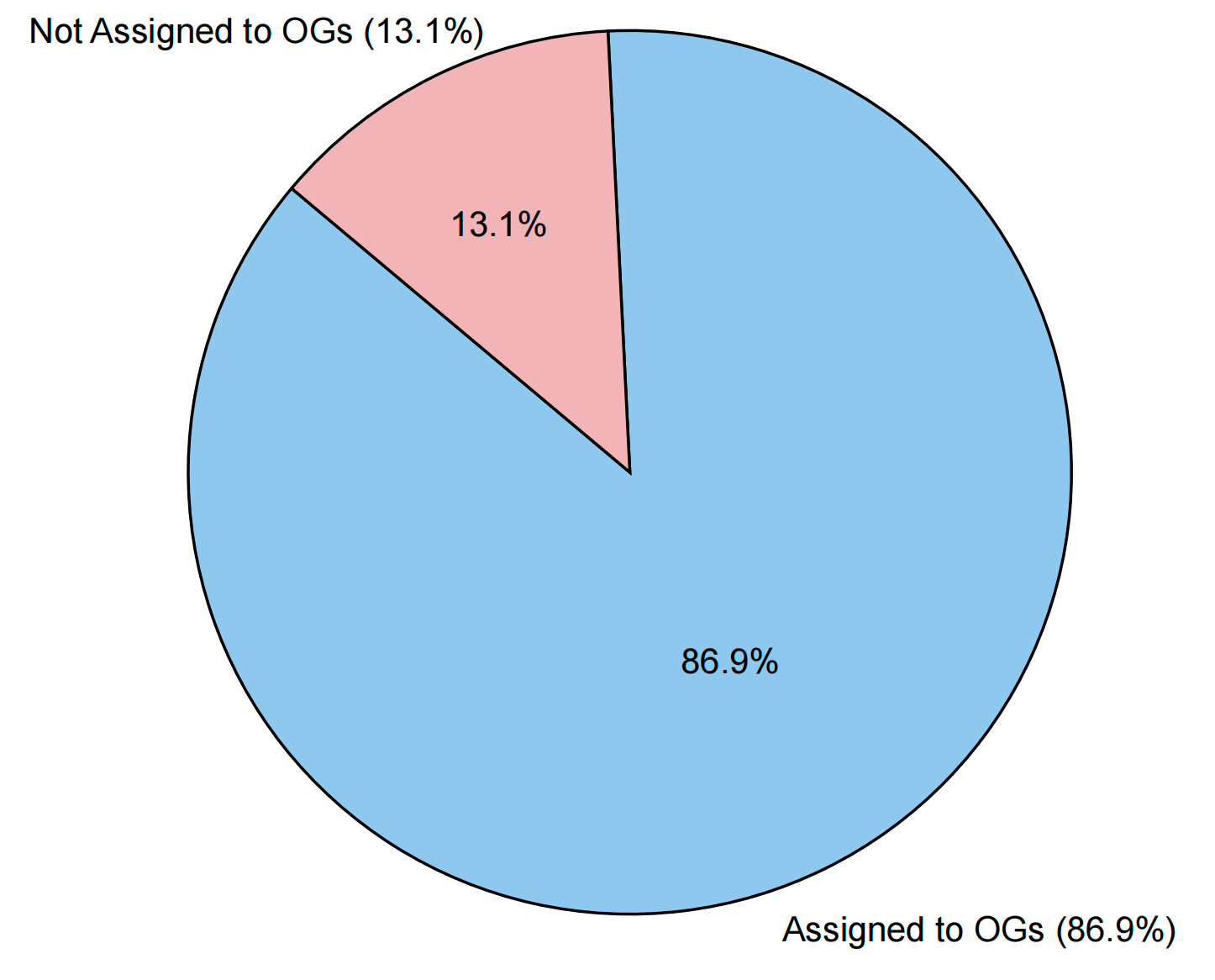


**Figure S3 Gene family clustering.** This figure shows the use of Orthofinder for gene clustering, with blue representing the proportion that can be classified into gene families and pink representing the proportion that has not been assigned to specific gene families.

**Figure S4.**


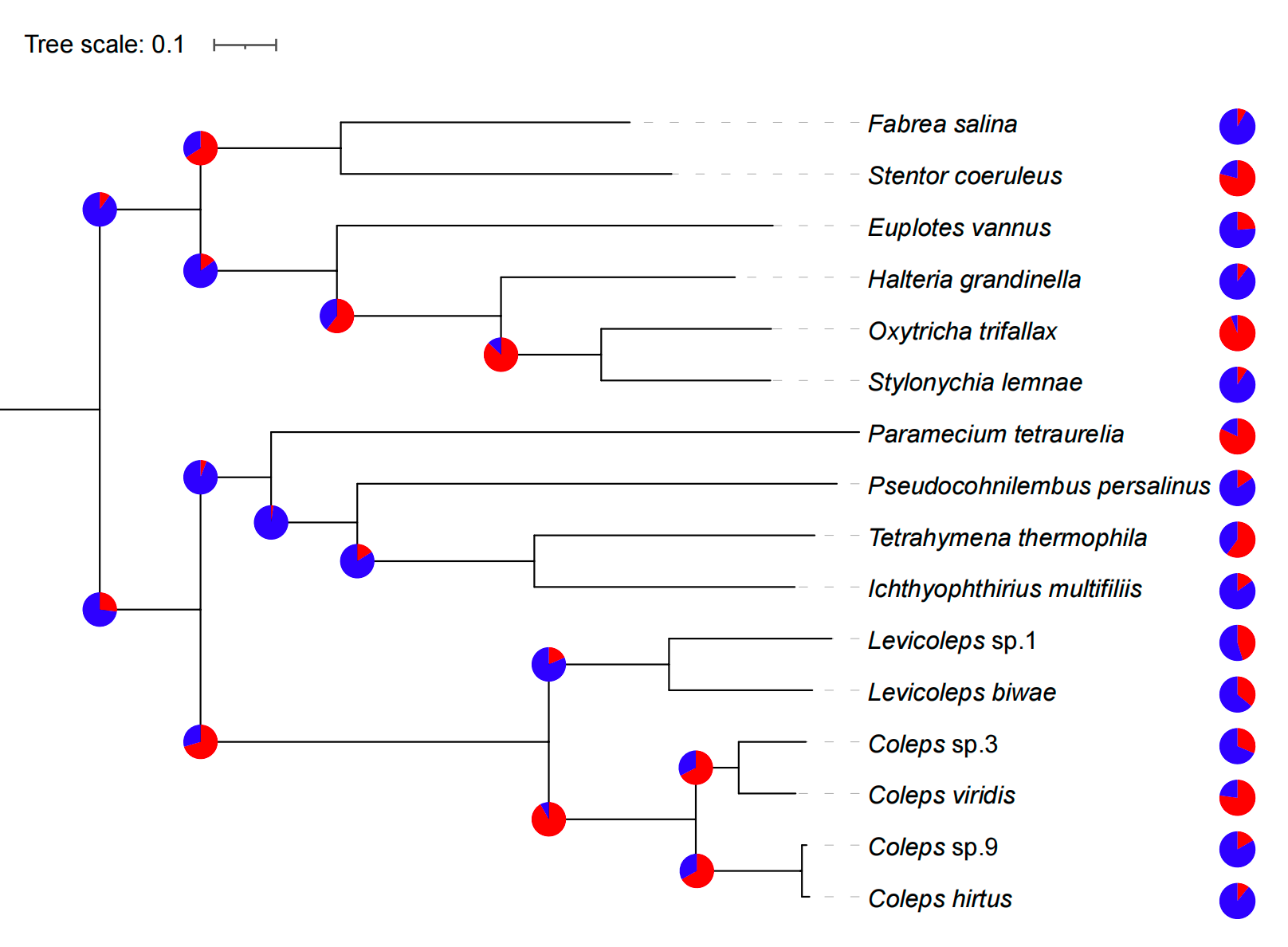


**Figure S4 Expansion and contraction analysis of the specific situation.** This figure specifically describes the proportion of contraction and expansion analysis results of 16 ciliates using CAFE analysis. Red represents the expansion gene family, and blue represents the contraction gene family.

**Figure S5.**


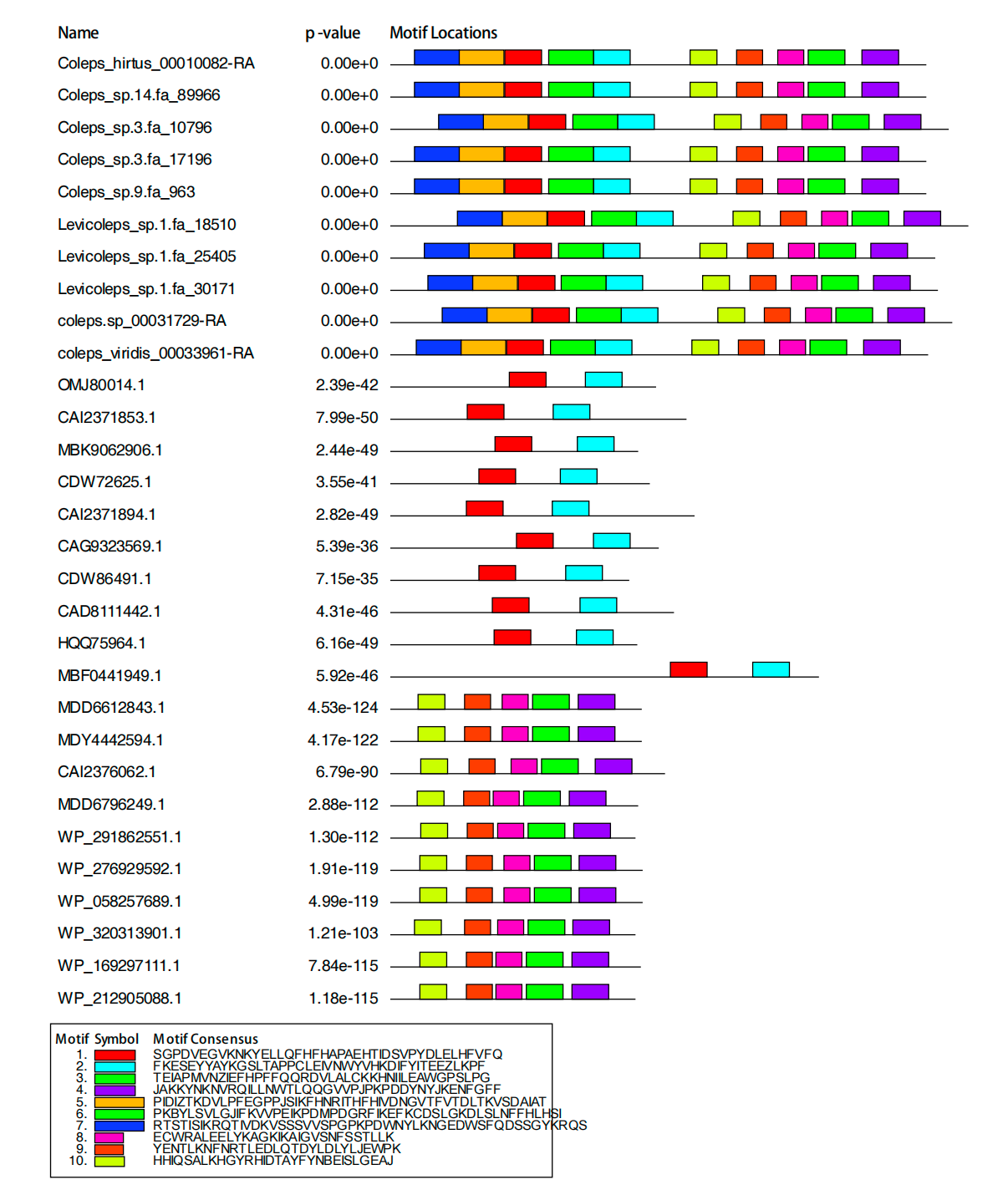


**Figure S5 Motif analysis of two-domain proteins.** This figure shows the motif analysis results of the 10 proteins that are most similar to the two domains of the dual domain protein.

**Figure S6.**


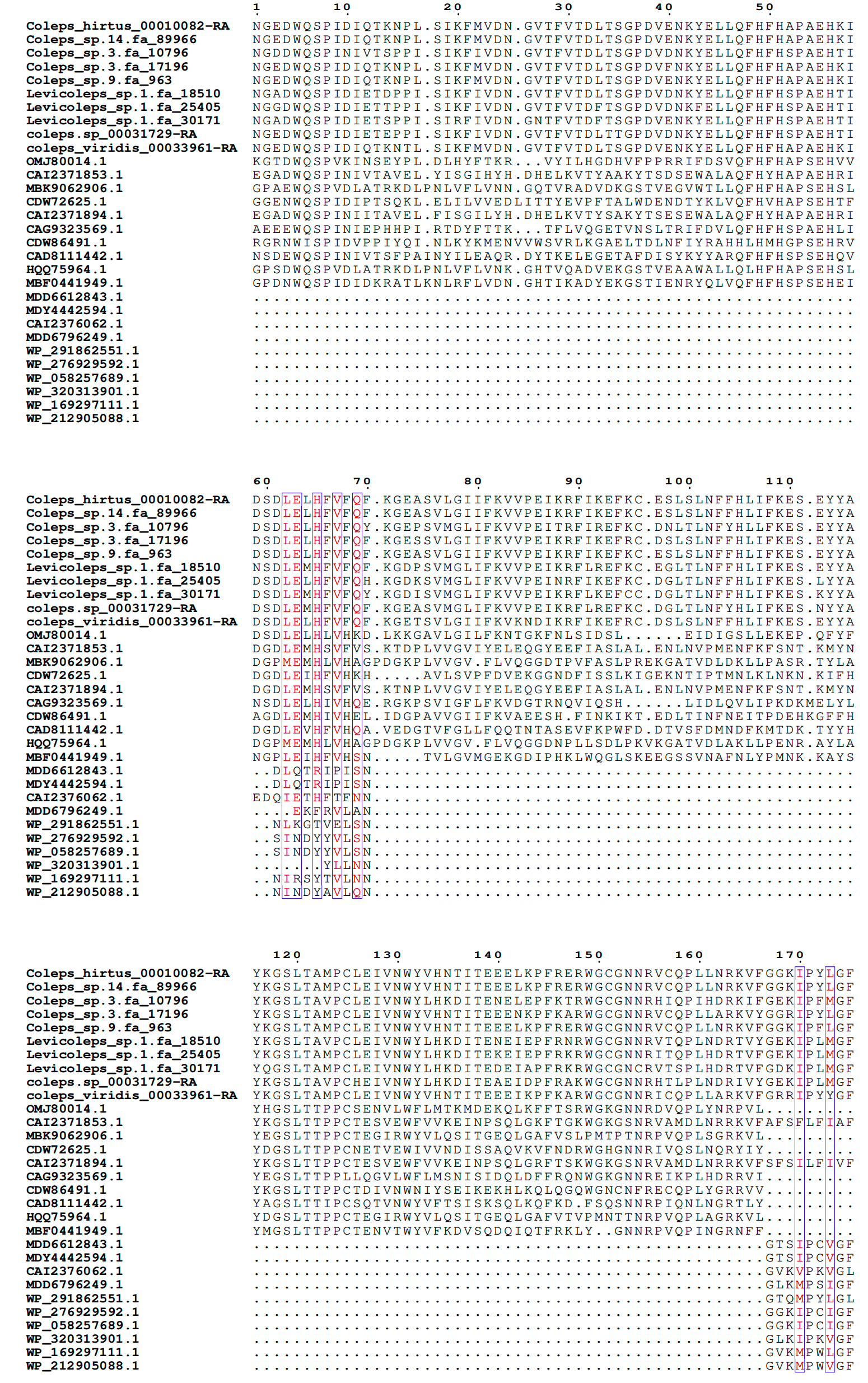


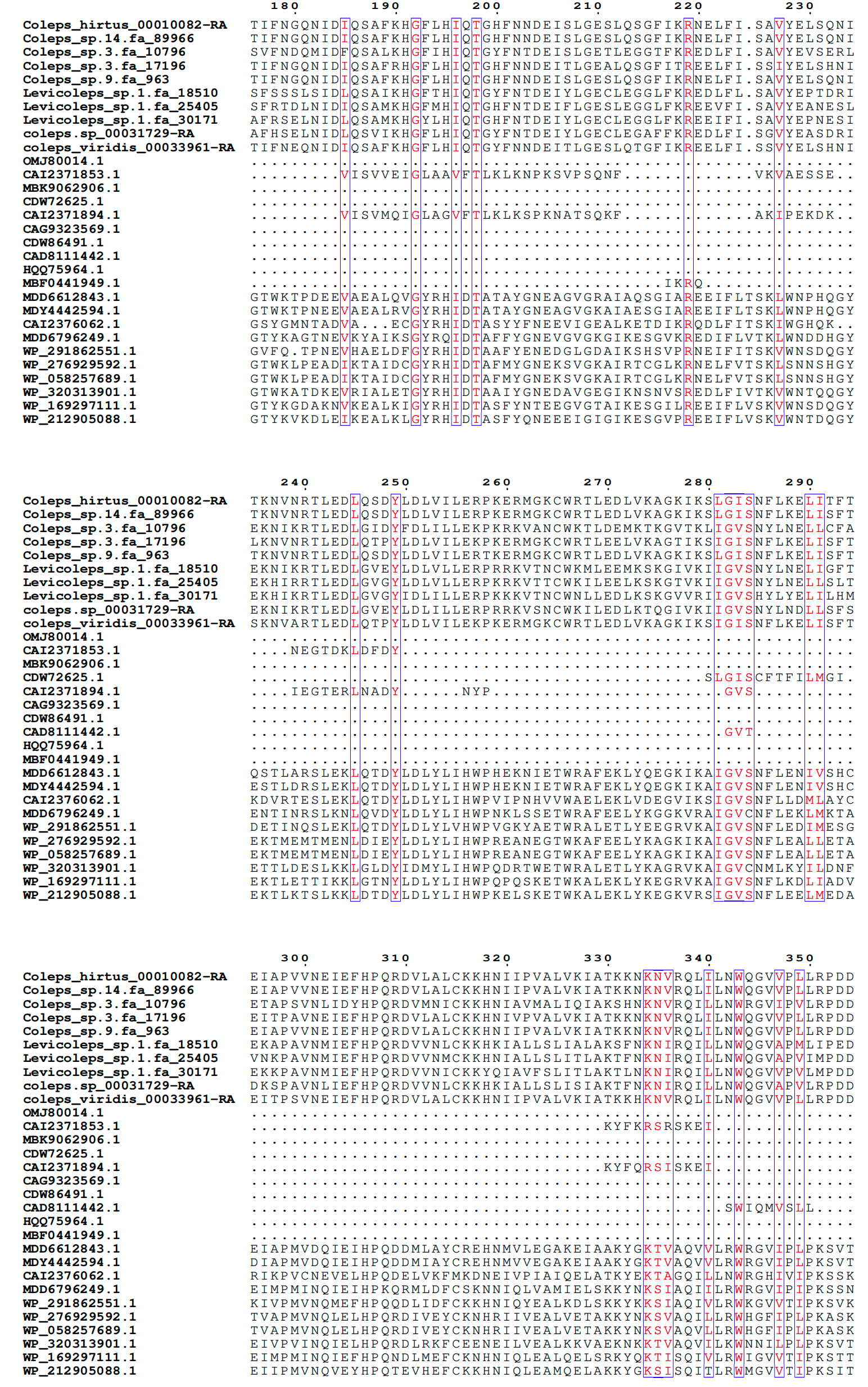

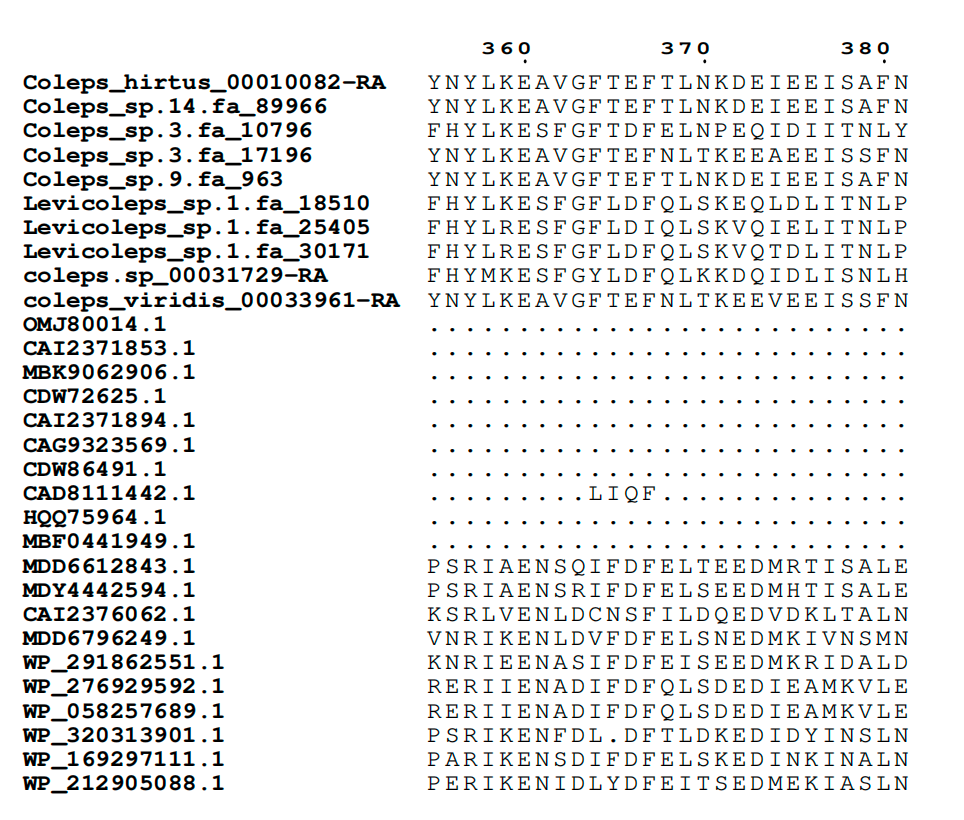


**Figure S6 Alignment of Carb::Aldo dual domain protein.** This Figure shows the comparison with Carb: Alignment of 20 similar proteins in the Aldo dual domain protein family comparison results

**Figure S7.**


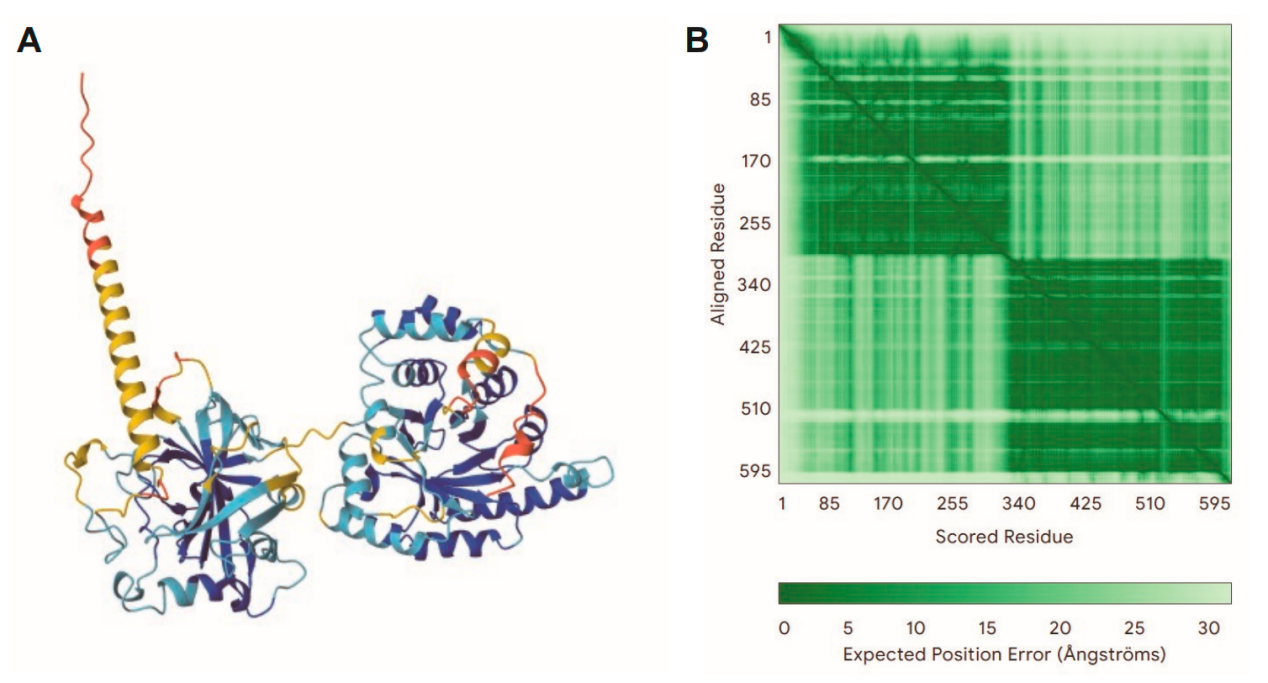


**Figure S7 Tertiary Structure prediction of Carb::Aldo dual domain protein.** A This figure shows the prediction of the tertiary structure of the Carb:: Aldo dual domain protein. Figure A shows the predicted three-level structure diagram, plotted in pLDDT quality color. B This figure shows the Expected Position Error of the predicted three-level structure.

**Figure S8.**


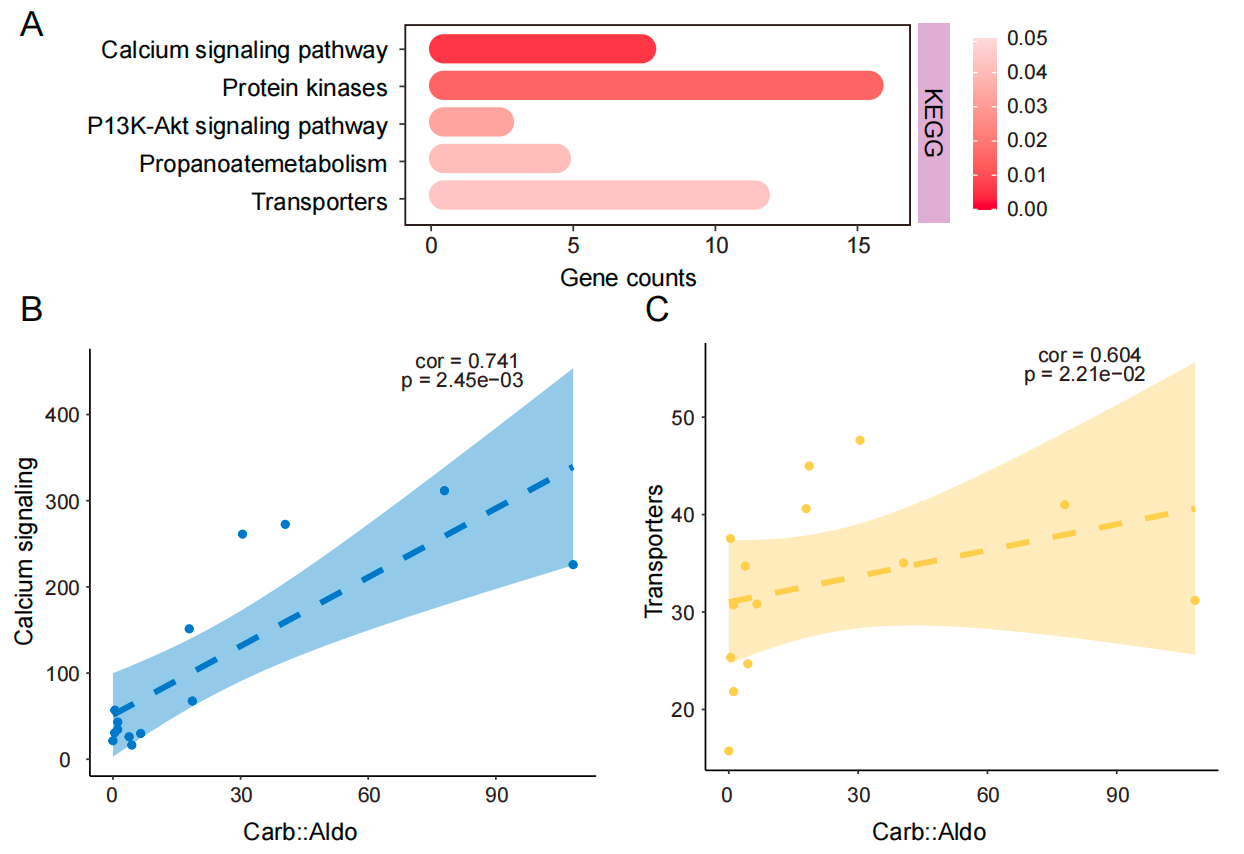


**Figure S8 KEGG Pathway Enrichment of Shared DEGs.** A The KEGG pathway enrichment analysis of shared differentially expressed genes (DEGs). B The correlation between the expression levels of the Carb::Aldo fusion gene and genes involved in the calcium signaling pathway and transporters pathway, calculated the Spearman correlation coefficients.

**Figure S9.**

**Figure S9 Experimental validation of *Carb::Aldo* Fusion Gene via phenotypic analysis and protein expression.** A Images of the length-to-width ratio for the two groups. B Armored individuals of *C. hirtus* cannibalized the unarmored conspecifics. C SDS-PAGE analysis of purified proteins. C1 *Carb::Aldo* fusion protein (~70 kDa), C2 *Carb* domain (~26.9 kDa); C3 *Aldo* domain (~31.1 kDa); Lane M: protein marker; Lane 1 arrowhead: purified target protein. Lane 2 Bovine Serum Albumin D Western Blot validation of purified proteins using anti-His tag antibody. M: prestained protein marker; D1 *Carb::Aldo* fusion protein (~70 kDa), D2 *Carb* domain (~26.9 kDa); D3 *Aldo* domain (~31.1 kDa); Lane 1 arrowhead: purified target protein. Lane 2 Multitag Protein.

**Table S1.**

**Table S1 Genomic Features of** **Three Colepidae Species**

| Species | *Coleps hirtus* | *Levicoleps biwae* | *Coleps viridis* |
| --- | --- | --- | --- |
| **Genome assembly** | | | |
| Total length(Mb) | 81.25 | 111.70 | 117.20 |
| Total length | 81,246,602 | 111,702,003 | 117,197,614 |
| Contigs | 10,258 | 9,236 | 25,554 |
| Largest contig | 182,821 | 482,011 | 145,139 |
| GC | 30.34% | 33.12% | 31.62% |
| N50 | 18,370 | 48,497 | 12,044 |
| N90 | 3,311 | 4,908 | 1,428 |
| **Annotation** | | | |
| Number of predicted gene | 25,088 | 34,981 | 42,772 |
| Genes annotated to protein domain | 23,635 | 32,752 | 40,012 |
| Genes annotated to KEGG database | 3,155 | 7,091 | 4,377 |
| DNA sequence mapping ratio | 81.63% | 92.28% | 78.07% |
| **completeness** | | | |
| Complete and single-copy BUSCOs | 84.80% | 90.10% | 86.50% |
| Complete and duplicated BUSCOs | 1.20% | 5.80% | 2.30% |
| Fragmented BUSCOs | 8.80% | 1.20% | 5.80% |
| Missing BUSCOs | 5.20% | 2.90% | 5.40% |
| Completeness of EUKCC | 94.81% | 98.70% | 98.70% |
